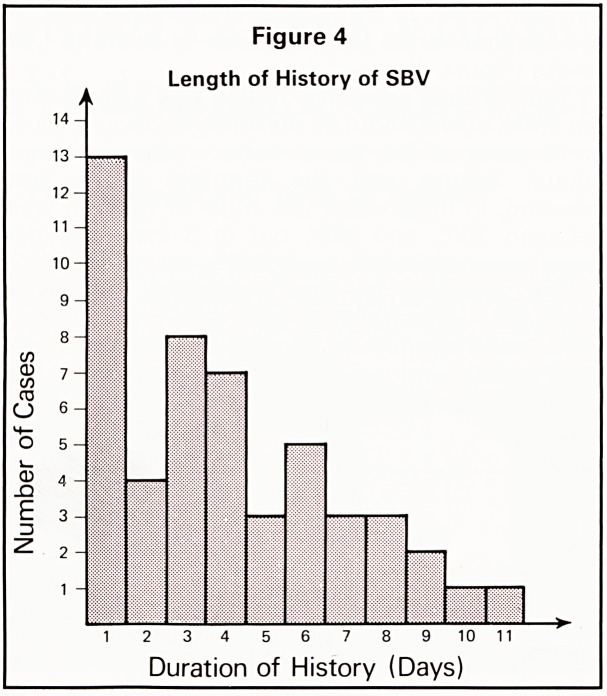# Small Bowel Volvulus — The Commonest Abdominal Emergency in Nepal

**Published:** 1980

**Authors:** I. O. McDonald, D. B. G. Hawkin


### Small Bowel Volvulus – The Commonest Abdominal Emergency in Nepal

**I. O. McDonald and D. B. G. Hawker**

*Shining Hospital, Pokhara, Nepal with Annapurna in the background.*

### INTRODUCTION

The Shining Hospital is situated in the town of Pokhara in the Himalayan foothills, at an altitude of 1000 metres. To the north lie the desolate peaks of the Annapurna range, while in other directions numerous small villages are scattered among the hills. The people of these agrarian communities, together with the traders of Pokhara Bazaar, make up the hospital's catchment population of about one million.

Operative surgery has been undertaken at the hospital since it was opened in 1953. The present series of cases covers the 20 years from 1957 to 1976, during which some 6000 operations, mainly on obstetric and gynaecological cases, or for trauma, were performed.

### METHODS

The inpatient notes and operation records of all patients who had undergone emergency abdominal surgery at the Shining Hospital between 1957 and 1976 were studied, and cases of small bowel volvulus (SBV) were analysed in detail. Cases of abdominal trauma, and obstetric and gynaecological emergencies were excluded.

### RESULTS

There were 192 emergency abdominal operations in the 20-year period, 130 of which were for intestinal obstructions. The age-distribution is shown in Figure 1. Cases are listed by diagnostic category in Table I. The majority of patients (72%) were male.

67 cases (34.9% of all emergencies, and 51.5% of intestinal obstructions) were SBV. 51 of these (76%) were male, and 16 female. The condition was found in all age groups, but most commonly in adult males (Figure 2) (Age range 4–67, m = 38).

A seasonal variation in incidence was noted (Figure 3) with a peak in the monsoon and a lesser peak in the winter.

SBV has been classified as 'primary' or 'secondary' according to the presence or absence of a predisposing lesion (Shepherd, 1968). Of the

**Figure 1**

**Age-Incidence of Abdominal Emergencies**

**Table I**

**Diagnostic Categories of Abdominal Emergencies**

| | *male* | *female* | *total* | *%* |
|---|---|---|---|---|
| small bowel volvulus* | 51 | 16 | 67 | 34.9 |
| external herniae | 26 | 6 | 32 | 16.7 |
| obstructions from adhesions | 8 | 12 | 20 | 10.4 |
| perforated peptic ulcer | 12 | 4 | 16 | 8.3 |
| other perforations | 7 | 8 | 15 | 7.8 |
| appendicitis | 13 | 0 | 13 | 6.8 |
| abdominal tuberculosis | 6 | 4 | 10 | 5.2 |
| malignancy | 4 | 0 | 4 | 2.1 |
| miscellaneous | 11 | 4 | 15 | 7.8 |
| | 138 | 54 | 192 | 100.0 |

* 1 case of appendicitis, 1 of malignancy, 2 cases of hernia and 2 of abdominal TB are listed under SBV, which was the principal diagnosis (see Table II).

**Figure 2**

**Age-Incidence of SBV**

present series, 44 (65.7%) were unequivocally primary, while an additional lesion was found in the remaining 23 (Table 2). No cases of volvulus neonatorum were seen, and in only one patient was there an obvious malformation of the mesentery.

The clinical presentation was similar to many obstructive conditions of the bowel, with vomiting, abdominal pain, constipation, and distension being the predominant symptoms. The pain was colicky and often sudden in onset, although some patients admitted to frequent bouts of vague abdominal pain in the past. In some cases of sub-acute onset the constipation was preceded by diarrhoea. On examination, the abdominal signs of bowel obstruction, with or without peritonitis and ascites, were frequently accompanied by dehydration and shock. The time between onset of symptoms and presentation at the hospital varied from a matter of hours to two weeks (Figure 4).

The principal operative finding was a rotation of the small bowel about its mesenteric-vascular axis. In 18 cases all the bowel between the duodeno-jejunal flexure and the terminal ileum was involved. In most cases the angle of rotation was between 180°, and 360°, but in 3 instances the bowel was rotated through 540°.

The twisting of the mesentery invariably produced strangulation of the mesenteric vasculature so that the affected bowel was purple from venous engorgement; in 9 cases (13.4%) the bowel was gangrenous.

**Figure 3**

**Seasonal Incidence of SBV**

Dates were recorded using the Nepali calendar, in which the New Year begins in mid-April.

**Table 2**

**Predisposing Conditions in Secondary Small Bowel Volvulus**

| *Condition* | *No. of Cases* |
|---|---|
| adhesions and abcesses | 7 |
| small bowel diverticula incl. Meckel's | 4 |
| abdominal tuberculosis | 2 |
| appendicitis | 2 |
| inguinal herniae | 2 |
| recent caesarian section (no adhesions) | 2 |
| caecal lymphoma | 1 |
| worm obstruction | 1 |
| torsion of ovarian cyst | 1 |
| traumatic perforation of gut | 1 |

**Figure 4**

**Length of History of SBV**

Blood-stained fluid was frequently found in the peritoneal cavity, and often the bowel itself was considerably distended by fluid in the lumen. In 18 patients (27%) the presence of parasitic worms was noted.

The operative treatment varied from simple derotation of the gut if it was viable, to resection of large portions of gangrenous bowel. Where distension of bowel was a marked feature, decompression was performed. Enteropexy was not attempted.

Sub-acute cases (length of history >24 hours) appeared to have a better prognosis than the acute fulminating cases (history ≤24 hours): 6 of the 13 acute cases (46%) died, while only 12 of the 54 sub-acute cases (22%) died.

The overall mortality was 26%.

Of the 13 cases for which the time of death was recorded, 10 died during the operation or in the following 24 hours. The greatest risk seemed to be associated with the moment of closure of the laparotomy incision.

In no case was the volvulus known to recur. Post-operative complications included two cases each of intestinal fistula, wound dehiscence, and aspiration pneumonia. Otherwise, prognosis was generally favourable for patients who survived the first post-operative day, and the mean time from operation to discharge from hospital was 16 days.

### DISCUSSION

The incidence of SBV varies greatly from country to country (Table III). There are few reports of substantial series from Western Europe and North America: Vick (1932) reported an incidence of 1.2% of intestinal obstructions in the UK, and from the USA the figure of 5.5% has been quoted (Waldron and Hampton 1961). In these series, no distinction was made between primary and secondary SBV, but the primary form is generally considered rare enough to warrant publication of individual cases occurring in the UK (Postlethwaite and Dupont 1973, Renton 1965, Walker 1960, Tagart 1950).

By contrast, in parts of Africa and Asia SBV is common. In Iran it accounted for 19.6% of intestinal obstructions (Vaez-Zadeh et al 1969), in Uganda 18.5% (de Souza 1976), and in Kenya 19.7% (Warambo 1971). According to Kerr and Kirkaldy-Willis (1946) SBV was the commonest

**Table 3**

**Reported Incidence of SBV in various countries**

| *Country* | *Author & Year* | *Period of Study* | *Total Obstructions* | *Total SBV* | *Primary SBV* |
|---|---|---|---|---|---|
| Afghanistan | Duke & Yar 1977 | 13 months | – | 26 | 26 |
| India | Gulati et al 1973 | 6 years | 1530 | 54 (3.5%) | 38 (2.5%) |
| Iran | Vaez-Zadeh et al 1969 | 5 years | 209 | 41 (19.6%) | 33 (15.8%) |
| Kenya | Kerr & Kirkaldy-Willis 1946 | 1 year | 21 | 7 (33.3%) | 6 (28.6%) |
| Kenya | Warambo 1971 | 5½ years | 142 | 28 (19.7%) | 18 (12.7%) |
| Nepal | present series | 20 years | 130 | 67 (51.5%) | 44 (33.8%) |
| Norway | Svane 1965 | 30 years | – | 4 | 2 |
| Russia | Spasokukozki 1909 | | 96 | 28 (29.0%) | 28 (29.0%) |
| Uganda | de Souza 1976 | 2 years | 65 | 12 (18.5%) | 12 (18.5%) |
| UK | Vick 1932 | 6 years | 6892 | 85 (1.2%) | – |
| USA | Waldron & Hampton 1961 | | 493 | 27 (5.5%) | – |

cause of acute intestinal obstruction in Kenya. In the Indian sub-continent there are wide variations between 3.5% in Delhi and 51.2% in Bihar (Gulati et al 1973, Banerji 1950). Not only does SBV account for a larger proportion of intestinal obstructions in these countries than in Western Europe or the USA, but the primary form accounts for a much larger proportion of the cases of SBV itself.

In this series from Nepal, the incidence of all cases of SBV (over half of intestinal obstructions), and of cases of primary SBV (over a third of intestinal obstructions) is remarkably high. Another unusual feature is that it occurred in both sexes in Nepal, and at all ages. Most series show a greater preponderance of adult males, and in Afghanistan, all the cases seen by Duke and Yar (1977) were in men.

Shock was a prominent feature in Nepal, as in Kenya (Warambo 1971). The local Nepali inclination to withhold fluids from the ill probably contributed to this. De Souza (1976) saw 'no general systemic manifestations of circulatory collapse' amongst his cases in Uganda, who had all been drinking heavily.

In most of our patients the clinical picture provided ample indication for urgent rehydration, nasogastric aspiration, and laparotomy. Radiology was usually unneccessary but the typical appearances are of distended loops of small bowel with multiple fluid levels on plain abdominal films. Occasionally more specific signs may be seen, such as jejunal mucosal pattern in the pelvis (Postlethwaite and Dupont 1973) or gas in the mesenteric vein without gas in the portal vein (Cynn and Hodes 1973). However none of these signs is pathognomonic of SBV, and an urgent laparotomy is usually required whatever the radiographic appearances.

The reported mortality varies from 11.6% (Duke and Yar 1977) to 57% (Andersen 1956). The mortality of 26% in Nepal is almost identical to that recently reported from India (Gulati et al 1973). Gangrene is generally regarded as a grave complication of SBV, (although it did not affect the prognosis in Gulati's patients). In Nepal, the mortality increased to 44% in the presence of gangrenous bowel. If the fluid obtained from a diagnostic peritoneal tap is blood-stained the bowel is probably necrotic (Warambo 1971) and operation is 'mandatory' (Moretz and Morton 1950).

The frequency of the epitaph 'died on closure' in the operation records of fatalities in this series emphasises the importance of adequate decompression of distended bowel. This measure reduces splinting of the diaphragm and pressure on the inferior vena cava to which, presumably, these patients succumbed.

**AETIOLOGY AND PATHOGENESIS:**

Presumably secondary SBV arises when an adhesion at the apex of a loop of bowel provides a fulcrum about which the loop can twist, given the right combination of forces from the diaphragm, the abdominal wall muscles, and the weight of a food bolus.

In primary SBV the cause is not obvious. Duke and Yar (1977) pointed to the tenfold increase in incidence among Moslems in Afghanistan during Ramadan, as evidence that dietary behaviour is involved: their patients had eaten large quantities of a coarse high-fibre diet after fasting all day. In Nepal where the population is largely Hindu, there is also a marked seasonal variation, with the peak during the monsoon – but this does not correspond with the Nepali festivals.

Vaez-Zadeh and colleagues (1969), developing the widely accepted ideas of Spasokukozki (1909), suggested that when a bulky bolus of food entered the proximal jejunum, that loop fell down into the pelvis, causing the empty distal small bowel to rise into the right upper quadrant. Rapid emptying of the stomach combined with the action of the diaphragm was said to cause the distal bowel to spread across into the left upper quadrant. As these distal loops themselves filled up they would fall into the left lower quadrant, completing a 360° twist.

This mechanism requires a long small bowel, a broad mesentery free of fat which might splint it, very firm abdominal muscles restricting bowel movements to the coronal plane, and an exceptionally high-bulk diet eaten rapidly on an empty stomach. All these criteria are met in the populations where primary SBV is prevalent. Also, the common history of frequent episodes of abdominal pain (Svane 1965, Renton 1965, Postlethwaite and Dupont 1973) and the common operative finding of fibrous thickening of the mesentry, or 'mesenteritis' (Popovici et al 1975, Vaez-Zadeh et al 1973, Gulati et al 1969) lend substance to the idea that partial volvulus had been a regular occurrence before it became irreducible.

Evidence against this mechanism is that:

(a) not all primary SBV is in the clockwise direction (de Souza 1976) which the theory would require;
(b) it does not account for further twists after the first 360°: some of our patients had a volvulus of 540° or more;
(c) solid or semi-digested food is not always found in the lumen (de Souza 1976, and our own cases) although Kerr and Kirkaldy-Willis (1946) found whole grain and maize husks in the small bowel of their patients;

(d) female patients, with lax abdominal muscles, developed primary SBV in Nepal.

Recently, evidence has been produced to suggest that the intrinsic motility of the gut is important in the production of volvulus.

(a) De Souza (1976) reported 12 cases from Uganda in which primary SBV had developed within a few hours of drinking large quantities of local beer containing high concentrations of 5-hydroxytryptamine which increases the motility of small bowel (Shepherd 1963).

(b) Freund (1976) described a case of primary SBV in a diabetic and suggested that changes in bowel tone and peristaltic activity resulting from diabetic autonomic neuropathy initiated the volvulus.

(c) Chronic parasitic infection of the bowel is rife in areas where primary SBV is prevalent, and was noted in many of our patients. Parasitism alters small bowel motility (Castro et al 1976). Chronic low-grade enteritis from worms, protozoa, or bacteria might alter the motile response of the small bowel to a bulky food bolus, initiating a volvulus (Gulati et al 1973).

While evidence exists to support the purely mechanistic explanation of primary SBV, it seems likely that a more complex process involving an alteration in gut motility mediated, perhaps, by chemicals in food or by low-grade enteritis, may have a part to play.

To date most studies on SBV have been retrospective. Even a simple prospective survey taking account of all the factors implicated in the aetiology, pathogenesis, and prognosis of primary SBV, would dispel much of the confusion surrounding the origin of this disease.

### REFERENCES

ANDERSEN, D. A. (1956) Volvulus in Western India, *Br.J.Surg.* **44**, 132.

BANERJI, B. N. (1950) Volvulus of small intestine. *Indian J.Surg.* **12**, 195.

CASTRO, G. A., BADIAL-ACEVES, F., SMITH, J. W. et al. (1976) Altered small bowel propulsion associated with parasitism. *Gastroenterology* **71**, 620–625.

CYNN, W.-S. and HODES, P. J. (1973) A new sign of small bowel volvulus. *Radiol.* **108**, 289–290.

DE SOUZA, L. J. (1976) Volvulus of the small bowel. *Br.Med.J.* **1**, 1055–1056.

DUKE, J. H. and YAR, M. S. (1977) Primary small bowel volvulus. *Arch.Surg.* **112**, 685–688.

FREUND, H. (1976) Volvulus of the small bowel in a diabetic patient. *Br.Med.J.* **2**, 641

GULATI, S. M., GROVER, N. K., TAGORE, N. K., et al (1973) Volvulus of the small intestine in India. *Am.J.Surg.* **126**, 661–664.

KERR, W. G. and KIRKALDY-WILLIS, W. H. (1946) Volvulus of the small intestine. *Br.Med.J.* **1**, 799–800.

MORETZ, W. H. and MORTON, J. J. (1950) Acute volvulus of the small intestine. *Ann.Surg.* **132**, 899–912.

POPOVICI, G., VINTILA, I., OLTEANU, C. (1975) Probleme de diagnostic si tratament in volvulusul de intestin subtire. *Rev.Chir.(Chir.)* **24**, 411–415.

POSTLETHWAITE, J. C. and DUPONT (1973) Two cases of idiopathic volvulus of the small bowel. *Br.J.Surg.* **60**, 479–480.

RENTON, C. J. C. (1965) Primary volvulus of small intestine. *Br.Med.J.* **2**, 743.

SHEPHERD, J. A. (1968) Surgery of the acute abdomen. 2nd ed. Edinburgh Livingstone, p.193–195.

SHEPHERD, J. J. (1963) Serotonin and gut motility. *Br.Med.J.* **2**, 1589.

SPASOKUKOZKI, S. (1909) Volvulus intestinorum als Krankheit des hungerden Menschen. *Arch.Klin.Chir.* **91**, 211.

SVANE, S. (1965) Volvulus of the entire small intestine in adults: four cases without anomalies of intestinal rotation. *Acta Chir.Scand.* **129**, 649–655.

TAGART, R. E. B. (1950) Volvulus of the small intestine: the position of relief. *Lancet.* **1**, 71–73.

VAEZ-ZADEH, K., DUTZ, W., NOWROOZ-ZADEH, M. (1969) Volvulus of the small intestine in adults: a study of predisposing factors. *Ann.Surg.* **169**, 265–271.

VICK, R. M. (1932) Statistics of acute intestinal obstruction. *Br.Med.J.* **2**, 546–548.

WALDRON, G. W. and HAMPTON, J. M. (1961) Intestinal obstruction: a half-century comparative analysis. *Ann.Surg.* **153**, 839–850.

WALKER, F. C. (1960) Primary volvulus of the small intestine in an adult. *Br.J.Surg.* **48**, 225.

WARAMBO, M. W. (1971) Acute volvulus of the small intestine. *East Afr.Med.J.* **48**, 209–212.

## Figures and Tables

**Figure f1:**
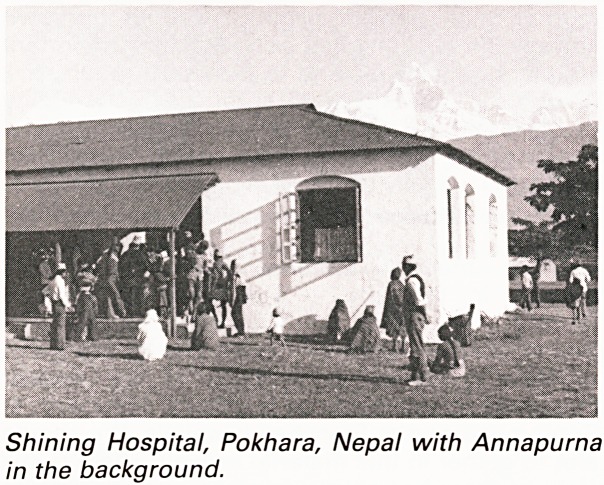


**Figure 1 f2:**
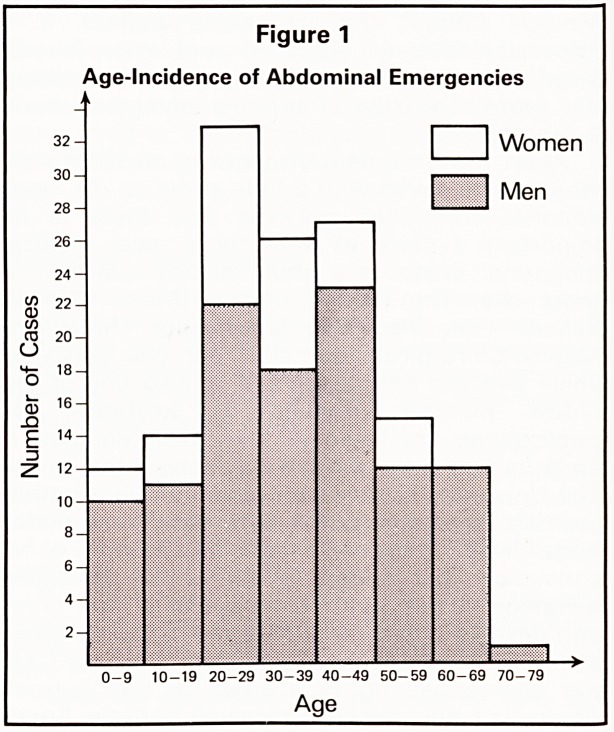


**Figure 2 f3:**
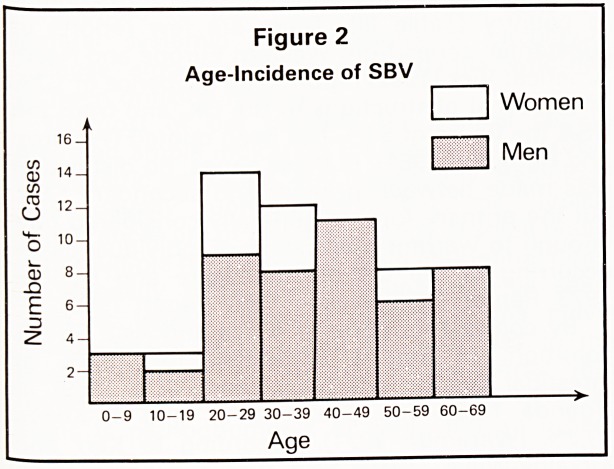


**Figure 3 f4:**
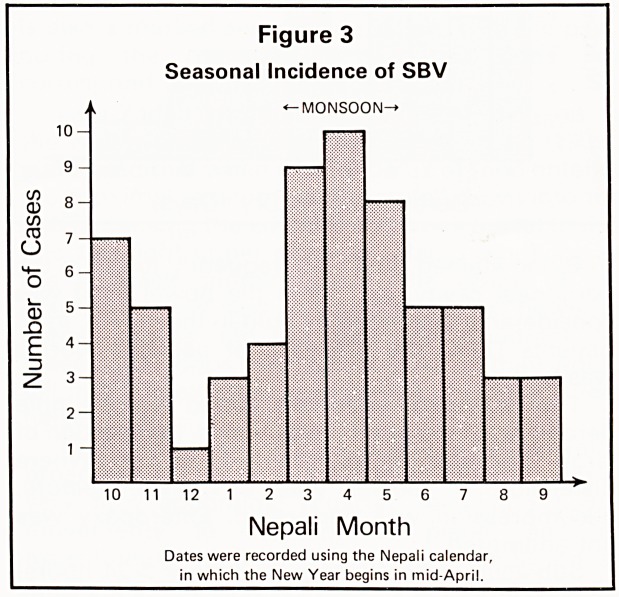


**Figure 4 f5:**